# A combined Langendorff-injection technique for simultaneous isolation of single cardiomyocytes from atria and ventricles of the rat heart

**DOI:** 10.1016/j.mex.2020.101189

**Published:** 2020-12-19

**Authors:** X.A. Butova, T.A. Myachina, A.D. Khokhlova

**Affiliations:** Institute of Immunology and Physiology Ural Branch of Russian Academy of science, Ural Federal University, Yekaterinburg Russia

**Keywords:** Cardiomyocyte isolation, Langendorff-based perfusion, Injection technique, Atrium, Ventricular free wall, Interventricular septum, Single cell mechanics, Calcium imaging

## Abstract

Single cardiomyocytes are widely used for investigations of the cellular and molecular mechanisms of regulation and modulation of cardiac performance. Intact cardiomyocytes allow one to study in detail cell function avoiding the effects of extracellular matrix and neighboring cells. The most established protocols of cardiomyocyte isolation are based on the isolated heart perfusion using a Langendorff-apparatus or on intraventricular perfusion using a syringe. However, the yield of single cardiomyocytes obtained by these methods may be low due to the cell injury following non-uniform enzyme digestion of connective tissue in different heart chambers. Moreover, isolation of atrial cardiomyocytes is challenging because of their small size and complex geometric shape. Here we present a new protocol for simultaneous isolation of high quality cardiomyocytes from the atria, ventricular free walls and interventricular septum. The protocol is based on the combination of the Langendorff perfusion method with the intraventricular and intra-atrial injection technique taking into account the collagen content variation between the different heart chambers. Obtained cells demonstrate rod-shaped morphology, a clear and regular sarcomere striation pattern and rat-specific frequency-dependence of contraction and calcium transient parameters. Our protocol provides gentle cell isolation that increases the yield of single cardiomyocytes suitable for biophysical researches .

## Graphical abstract

**Figure:**
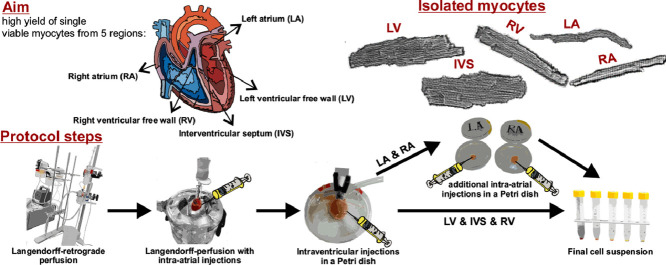

Specifications table

**Table:** 

Subject area	Medicine and dentistry
More specific subject area	Single cell biophysics, single cell biomechanics
Method name	Langendorff-injection
Protocol name	Combined “Langendorff-injection” technique
Reagents/tools	Reagents: • NaCl (Acros Organics, Belgium); • KCl (Acros Organics, Belgium); • KH_2_PO_4_ (Acros Organics, Belgium); • NaHCO_3_ (Acros Organics, Belgium); • NaOH (Acros Organics, Belgium); • Adenosine (Acros Organics, Belgium), • MgSO_4_ (Sigma Aldrich, USA); • CaCl_2_ solution (1 M in H_2_O) (Sigma Aldrich, USA); • EGTA (Sigma Aldrich, USA); • HEPES (Sigma Aldrich, USA); • D-Glucose (Sigma Aldrich, USA); • Taurine (Sigma Aldrich, USA); • Bovine Serum Albumin (Sigma Aldrich, USA); • Protease XIV~3.5 U/mg (Sigma Aldrich, USA); • Collagenase Type 2 ~305 U/mg (Worthington, USA); • Heparin Sodium 5000 IU/mL (Ellara, Russia); • Xylazine 2% (Alfasan, Netherlands); • Zoletil-100 (Virbac, France); • O_2_ (95%) / CO_2_ (5%) mixture gas. Laboratory equipment and instruments: • Electronic Balance Pioneer (Ohaus, USA); • pH-meter Starter 2100 (Ohaus, USA); • Langendorff-apparatus (custom-made); • Peristaltic pump (MasterFlex, USA); • Thermostatic water bath (IKA® ICC basic, Germany); • Magnetic stirrer with hotplate MR Hei-Standard (Heidolph Instruments, Germany); • Centrifuge CM-6MT (ELMI, Latvia) • Petri dish with aortic stand and steel hooks to fix the heart (custom-made) • 5 small Petri dishes (to collect separately tissue from the left and right atria, left and right ventricular free walls and interventricular septum); • Big and small scissors; • 2 fine precision tweezers with curved tips; • Alligator clips and ligature thread; • 4 insulin syringes (1 for intra-atrial injection during retrograde perfusion on the Langendorff-apparatus, 1 for the intraventricular injection in the custom-made Petri dish; 2 for separate injection into the left and right atria); • Mesh for single cell filtration
Experimental design	The protocol consists of 6 steps: Step 1: the preparation of the heart for perfusion; Step 2: retrograde heart perfusion using a Langendorff-apparatus; Step 3: combined Langendorff-injection perfusion; Step 4: intraventricular injections; Step 5: separations of the heart chambers and additional intra-atrial injections; Step 6: preparation and collection of the single cell suspensions.
Trial registration	None
Ethics	Directive 2010/63/EU and local Institutional Ethics Committee (Institute of Immunology and Physiology, Ural branch of Russian Academy of Science)
Value of the Protocol	1. The new protocol provides the gentle and reliable method for simultaneous isolation of highly viable intact cardiomyocytes from the atria, ventricular free walls and interventricular septum suitable for biophysical and biomechanical studies. 2. Our isolation protocol takes into account the collagen content variation between the different heart chambers that enables to significantly increase the cell yield; 3. The combination of Langendorff-based and Langendorff-free methods allows one to keep the advantages of each technique providing greater productivity.

## Description of protocol

### Rationale

An isolated cardiac myocyte is the essential research object to study in detail the intrinsic cellular and sub-cellular mechanisms of cardiac output regulation. The most established protocols of single cardiomyocyte isolation are based on the standard Langendorff retrograde perfusion [1], [2], [3] or simplified intraventricular injection technique [3], [4], [5], [6]. However, these methods do not take into account the variation in the connective tissue content between the heart chambers [7], [8], [9], which affects the yield of obtained viable cells. Here, we present an alternative protocol of simultaneous isolation of highly viable single cardiomyocytes from atria, ventricular free walls and interventricular septum using the combination of retrograde Langendorff-based perfusion and the intraventricular and intra-atrial injection technique with varying concentration of enzyme solution.

**Fig. 1:**
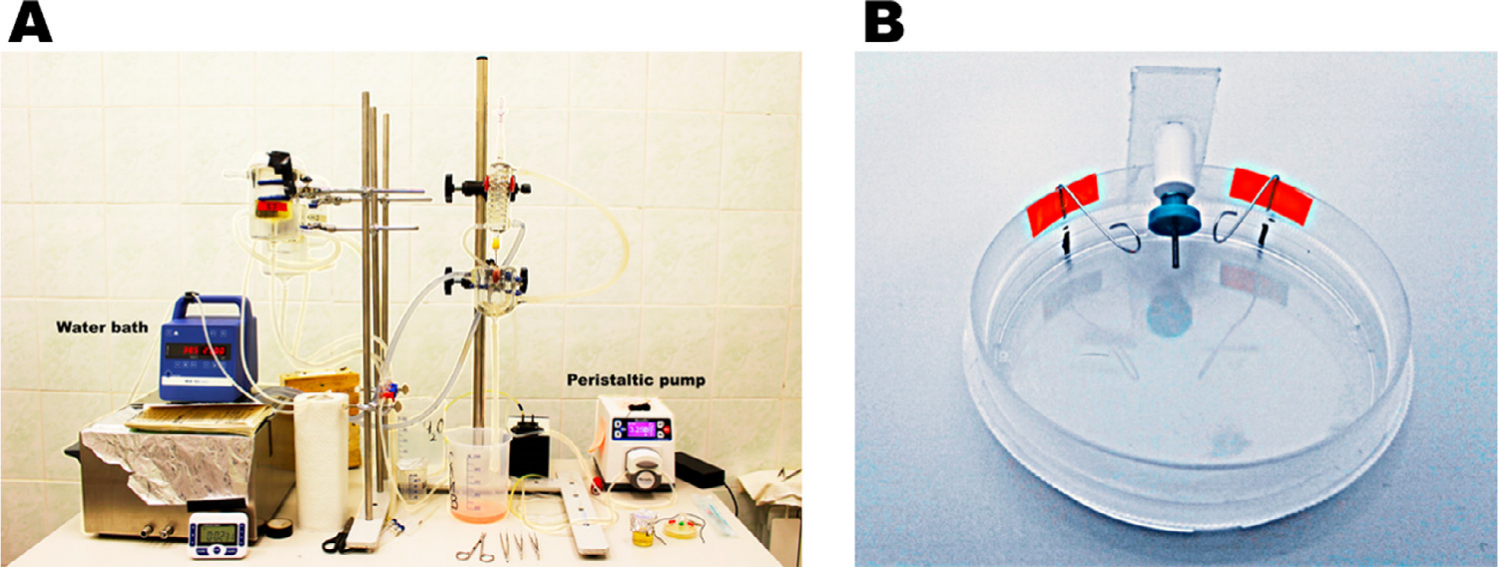
A Langendorff-apparatus (A) for retrograde perfusion and a custom-made Petri dish with aortic stand and steel hooks (B) for the injection technique.
• Peristaltic pump (MasterFlex, USA);
• Thermostatic water bath (IKA® ICC basic, Germany);
• Magnetic stirrer with hotplate MR Hei-Standard (Heidolph Instruments, Germany);
• Centrifuge CM-6MT (ELMI, Latvia)
• Petri dish with aortic stand and steel hooks to fix the heart (custom-made) (Fig. 1B)
• 5 small Petri dishes (to collect separately tissue from the left and right atria, left and right ventricular free walls and interventricular septum);
• Big and small scissors;
• 2 fine precision tweezers with curved tips;
• Alligator clips and ligature thread;
• 4 insulin syringes (1 for intra-atrial injection during retrograde perfusion on the Langendorff-apparatus, 1 for intraventricular injection in the custom-made Petri dish; 2 for separate injection into the left and right atria);
• Mesh for single cell filtration

Laboratory equipment and instruments:

• Electronic Balance Pioneer (Ohaus, USA);
• pH-meter Starter 2100 (Ohaus, USA);
• Langendorff-apparatus (custom-made) (Fig.1A);

## Reagents

### Procedure

All experiments were carried out according to the Directive 2010/63/EU and approved by Animal Care and Use Committee of the Institute of Immunology and Physiology of RAS.. Adult Wistar rats, aged 3–6 months, are maintained under standard conditions before their use in experiments. The rats are injected intramuscularly with heparin sodium (5000 IU/kg) to prevent development of coronary thrombosis, anesthetized 20 min later with Zoletil (0.3 mL/kg) and Xylazine (1 mL/kg) and euthanized in 15–20 min.

### Step 1 – Preparation of the heart for perfusion

After euthanasia, the chest is rapidly opened, and the heart is flashed with heparin-containing cold (10–15 °C) K-H buffer (Table 1). Then the heart is immediately removed together with the lungs and placed in cold K-H buffer. Note, that it is important to do not cut completely the descending aorta, pulmonary trunk, as well as the venae cavae and pulmonary veins, to be able to easily change the heart pressure (see Step 2). We also do not remove the pericardium to prevent possible ischemic effects during the heart stabilization on a Langendorff-apparatus.

**Table 1:** Krebs-Henseleit bicarbonate buffer (K-H buffer).

Compound	Molar Weight (g/M)	Final concentration (mM)
NaCl	58.44	121
KCl	74.55	4.7
MgSO_4_	120.37	1.2
KH_2_PO_4_	136.09	1.2
NaHCO_3_	84.01	19.0
HEPES	238.30	10.0
Taurine	125.15	20.0
Adenosine	267.24	2.0
d-Glucose	180.20	11.1
CaCl_2_	110.98	1.0

pH 7.25 with NaOH at 35°C.

95% O_2_ + 5% CO_2_ for 30 min before pH calibration.

After pH calibration add heparin sodium (10 IU/ mL).

### Step 2 – Retrograde heart perfusion using a Langendorff-apparatus

**Fig. 2:**
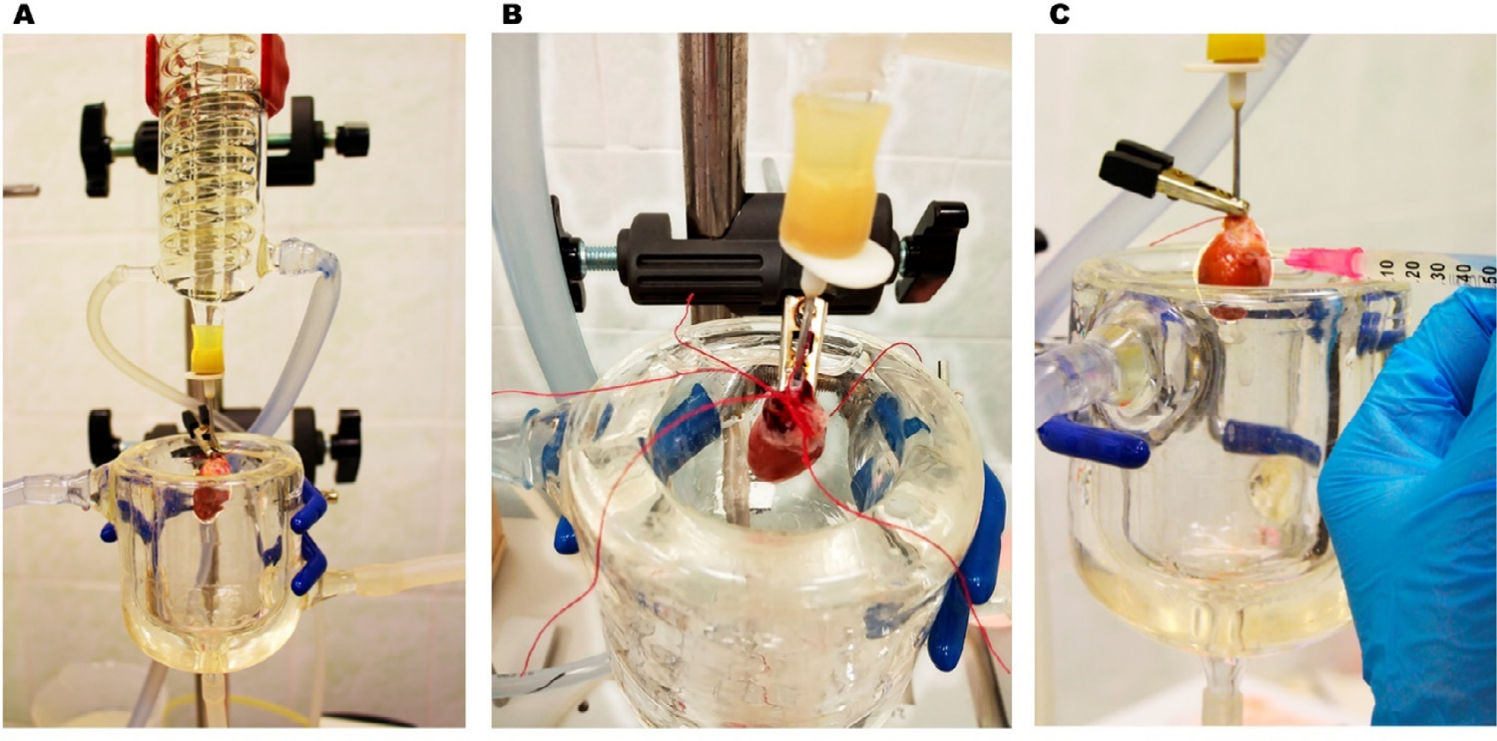
Representative illustration of the protocol steps based on the Langendorff method. (A). The heart mounted on a Langendorff perfusion apparatus when the aorta is cannulated, fixed with an alligator clip and tied with the ligature thread (step 1). (B). The ligation of the pulmonary trunk and inferior vena cava (step 2). (C). The injection of the left and right atria with a High-concentrated Enzyme solution (step 3).

The heart is swiftly transferred to a switched-on Langendorff perfusion apparatus and the aorta is cannulated (<30 s), fixed with an alligator clip and tightly tied with the ligature thread to the cannula. Perfusion is conducted at 35.5 °C at a rate of ~ 4.0–4.5 mL/min (depends on the heart size) with a sequence of three solutions (Tables 1–3) equilibrated with 95% O_2_+5% CO_2_ (Fig. 2). Perfusion is started with heparinized K-H buffer for 5 min after the heart has started normal beating (80–110 beat/min) to wash out from blood and maintain the isolated heart *in vitro* conditions. During perfusion with K-H buffer, the pulmonary trunk and veins are not ligated to prevent pressure overload (Fig. 2A).

**Table 2:** Low Ca^2+^ - high K^+^ Krebs-Henseleit bicarbonate buffer, Low-concentrated Enzyme solution and Stopping buffer.

Compound	Low Ca^2+^ - high K^+^ K-H buffer	Enzyme solution (Low-concentrated)	Stopping buffer
NaCl (mM)	105	105	105
KCl (mM)	14.7	14.7	14.7
MgSO_4_ (mM)	5	5	5
KH_2_PO_4_ (mM)	1.2	1.2	1.2
NaHCO_3_ (mM)	10.0	10.0	10.0
HEPES (mM)	10.0	10.0	10.0
Taurine (mM)	20.0	20.0	20.0
Adenosine (mM)	2.0	2.0	2.0
d-Glucose (mM)	11.1	11.1	11.1
EGTA (mM)	0.15	—	—
CaCl_2_ (mM)	0.05	0.025	0.025
Collagenase type II (mg/mL)	—	0.6	—
Protease XIV (mg/mL)	—	0.06	—
Bovine Serum Albumin (mg/mL)	—	—	5
pH with NaOH at 35°C 95% O_2_ + 5% CO_2_ for 30 min before pH calibration	7.0	7.25	7.25

**Table 3:** Enzyme solutions.

	Collagenase type II ~305 U/mg	Protease XIV ~3.5 U/mg
Low-concentrated	0.6 mg/mL	0.06 mg/mL
High-concentrated	0.8 mg/mL	—
Medium-concentrated	3 Low: 1 High

If the coronary flow and heart beating are sufficient, perfusion is switched to low Ca^2+^- high K^+^ K-H buffer (Table 2) for 10 min after the heart has stopped beating. When the heart is completely stopped the perfusion flow rate should be slightly increased (up to 4.5–5.0 mL/min) and the pulmonary trunk and a vena cava should be ligated to enhance the flow and intra-atrial pressure (Fig. 2B). The hallmarks of sufficient atrial perfusion are an increase in their size (a right atrium is usually larger than the left one) and a change in their color from deep to pale. case if the right atrium is swollen due to the pressure overload, the ligature is removed or the right atrium surface is slightly incised.

### Step 3 – Combined Langendorff-injection perfusion

Next, the heart is perfused with a Low-concentrated Enzyme solution for 10–15 min (Table 2) for enzymatic disruption of intercellular contacts at the intercalated discs. The perfusion flow rate should be again slightly increased (up to 5.2–5.5 mL/min). In 2–2.5 min later each atrium is injected (0.5–1 mL/min) with a High-concentrated Enzyme solution (Table 3) using an insulin syringe (Fig. 2C) during continuous retrograde perfusion. Tominimize the mechanical interventions and maintain optimal intra-atrial pressure, all injections should be performed at the same puncture site.

Then the Langendorff-perfusion should be stopped at the half-digested state. This state is determined by the pale color of the heart surface, relatively soft heart surface with preserved elasticity (check it by pushing), and, most important, by the appearance of viscous drops at the apex. In this stage, the heart should be rapidly transferred to a custom-made Petri dish (see laboratory equipment and instruments) with a warm (35° C) Medium-concentrated Enzyme solution (Table 3) equilibrated with 95% O_2_+5% CO_2_.

### Step 4 – Intraventricular injections

Then the heart is transferred to a Petri dish to perform further perfusion by a syringe. This approach provides gentle manually controlled digestion of the myocardium from all the chambers.

**Fig. 3:**
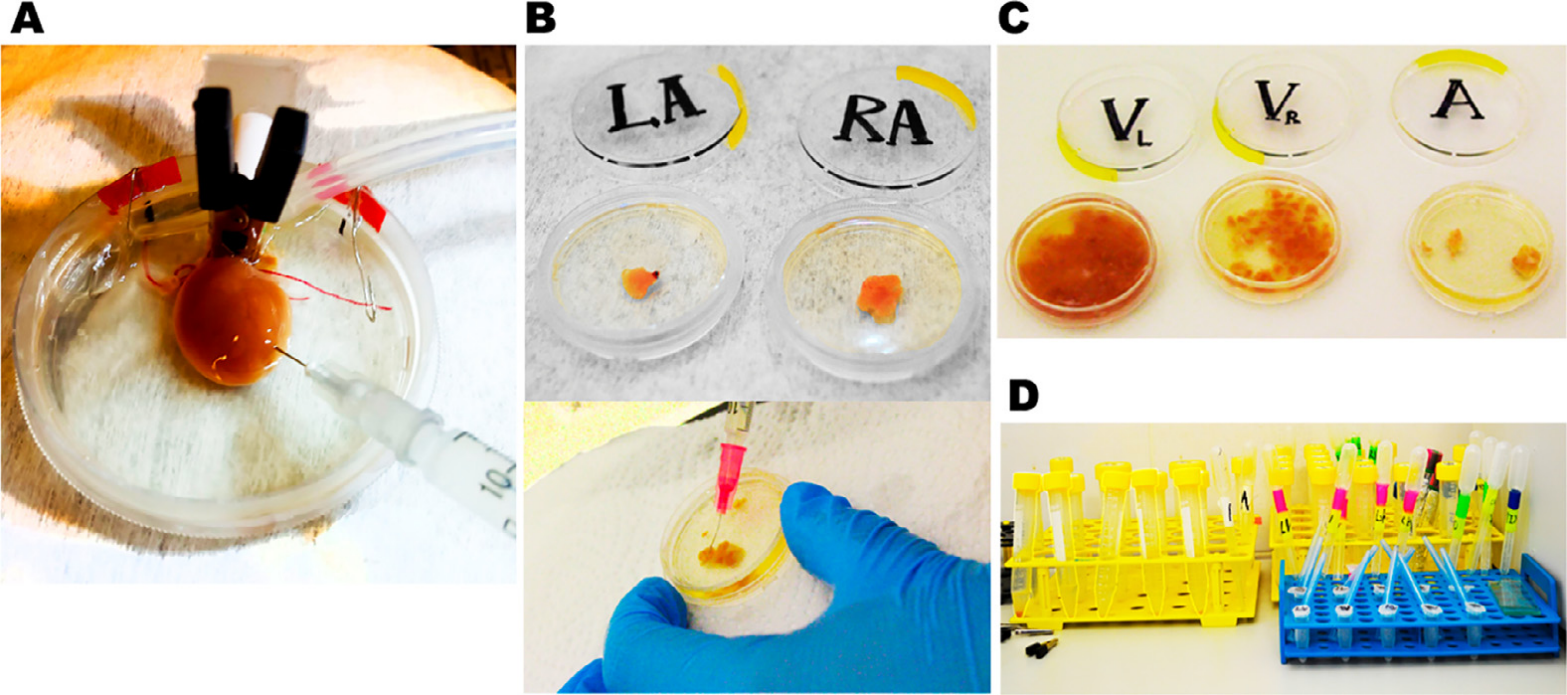
Representative illustration of the Langendorff-free injection method. (A). Intraventricular injections (step 4). (B). Intra-atrial injections (step 5). (С). Tissue disaggregation (step 6). Note the difference in the final structure of disrupted tissue between the left and right ventricles and the atria. (D). Sedimentation of cell suspensions (step 6).

In a custom-made Petri dish, the ascending aorta is fixed to the aortic stand by an alligator clip while the descending aorta and veins are ligated (to mountain optimal perfusion pressure) and fixed with special hooks. To maintain a stable temperature (35.5 °C) of the solution in a Petri dish we put it on the hotplate (Fig. 3). In this step, firstly, the heart is perfused only by injections of the left ventricular apical region using an insulin syringe (7 min, 5–6 mL/min). As previously, all injections should be performed at the same puncture site (Fig. 3A). Then the right ventricle is additionally perfused by injections into the apical region of the right ventricular free wall (12–15 min). The interventricular septum is perfused when the syringe needle is inserted completely into the septum (2–3 min). As previously, all injections should be performed at the same puncture site (Fig. 3A). It is also important to check that atria gradually increase in their size with each injection. The intraventricular perfusion is stopped when the signs of complete ventricular digestion are manifested. It includes a decrease in tissue resistance to injection flow, soft and glossy epicardial surface, a characteristic smell, the appearance of holes and extensive pale areas at the epicardium, and the appearance of single myocytes in the solution. 95% O2 + 5% CO2 was continuously supplied to the solutions during the entire procedure.

### Step 5 – Separations of the heart chambers. Intra-atrial injections

After complete digestion of the ventricles, the ligatures are removed and the heart is separated into 5 regions: the left and right ventricular free walls, interventricular septum, left and right atrium, each of which is quickly transferred in a separate Petri dish. Petri dishes with the left ventricular free wall and interventricular septum contain a Low-concentrated Enzyme solution with Stopping buffer (1:1, Table 2) To provide better digestion of the heart regions that have been less perfused, a Petri dish with the right ventricular free wall contains a Low-concentrated Enzyme solution and Petri dishes with atria contain a High-concentrated Enzyme solution without adding Stopping buffer.

Then, the ventricular free walls and interventricular septum are exposed to gentle mechanical disruption, re-suspended with Stopping buffer, filtrated and gradually adjusted to extracellular Ca^2+^ concentration (see Step 6). The left and right atria, which contain a greater concentration of collagen than ventricles are exposed to additional enzymatic digestion by intra-atrial injections with a High-concentrated Enzyme-solution for 20–24 min using separate syringes (Fig. 3B). Notably, the atria should not be completely hermetic. The atrioventricular septum was cut but intra-atrial perfusion flow (6–7 mL/min) was sufficient to provide optimal digestion. After the signs of the complete digestion of the atria are appeared (the same as for the ventricles, see above), the perfusion is stopped, and the atria are ready for mechanical disruption.

### Step 6 – Preparation and collection of single cell suspensions

Completely digested atria, free ventricular walls and interventricular septum are accurately disrupted using small scissors (Fig. 3C). A solution, containing ventricular cells, is filtered through a mesh, re-suspended with Stopping buffer (Table 2) and harvested by gravity sedimentation for 10–12 min. We do not filter the atrial cell suspension centrifuging it instead in Stopping buffer for 2 min at 7 × g (200 rpm) before gravity sedimentation. The sedimentation is repeated several times, while extracellular Ca^2+^ concentration is gradually increased to physiological values (1.8 mM CaCl_2_) (Table 4). The final cell pellet is stored in Hepes-buffered Tyrode solution (Table 5) at room temperature (22–24 °C). To prevent cell-to-cell adhesion the tubes with cell suspensions are stored at a 45° angle (Fig. 3D).

**Table 4:** A gradual increase in Ca^2+^ concentration.

Step	Solution	Calcium concentration
Step 0	Stopping buffer and Medium-concentrated Enzyme solution (1:1)	25 µM
Step 1	Stopping buffer	0.10 mM
Step 2	Stopping buffer	0.25 mM
Step 3	Stopping buffer	0.40 mM
Step 4	Stopping buffer	0.90 mM
Step 5	Hepes-buffered Tyrode	1.8 mM

**Table 5:** Hepes-buffered Tyrode.

Compound	Molar Weight (g/M)	Final concentration (mM)
NaCl	58.44	140
KCl	74.55	5.4
MgSO_4_	120.37	1
HEPES	238.30	10.0
d-Glucose	180.20	11.1
CaCl_2_	110.98	1.8

pH 7.35 with NaOH at 36–37 °C.

### Protocol validation

#### Measurements of the functional characteristics

To evaluate the viability and functional ability of isolated cardiomyocytes, we recorded sarcomere shortening and cytosolic free calcium transient using the LSM 710 laser confocal scanning microscopy system and Zen 2010 software (Carl Zeiss, Germany).

For calcium transient recordings, cells from each region were incubated in Tyrode solution supplemented with 2.5 µM calcium sensitive dye Fluo-8 AM (AAT Bioquest, USA) and 0.1% Pluronic® F-127 (AAT Bioquest, USA) in darkness for 20 min at room temperature. Before the measurements, cells were re-suspended in a dye-free Tyrode solution. An argon laser with a wavelength of 488 nm was used to excite the fluorescent dye. The emitted fluorescence was collected every 1–3 ms in the continuous scanning mode at 493–575 nm in a narrow (3 pixels high, 200–500 pixels long) region horizontally oriented along the long axis in the center of a cardiomyocyte. The change in fluorescence intensity (ΔF/F_0_, where F_0_ is the initial fluorescence) was calculated and used as an index of the change in cell cytosolic calcium concentration using custom-made software EqapAll 6 [10].

To record unloaded sarcomere shortening, the image-intensity profile in the selected narrow region was obtained in the optical channel. The method of Discrete Fourier Transform of the striation profile signal was used to determine the average sarcomere length in a cardiomyocyte using EqapAll6. The measurements were carried out after the cells reached a steady-state at an electric stimulation frequency of 1, 2, 3 and 4 Hz at a temperature of the Tyrode solution in the perfusion chamber of 36–37 °C.

**Fig. 4:**
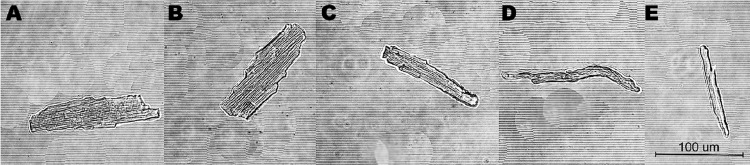
Representative images of single cardiomyocytes. (A). From the left ventricular free wall. (B). From the interventricular septum. (C). From the right ventricular free wall. (D, E). From the left and right atria. Magnification 40x. Scale bar=100 µm.

The yield of intact viable cells that can be achieved with our protocol is ≈ 60–90% for ventricular myocytes and ≈ 40–70% for atrial myocytes that are suitable for various measurements required the use of fluorescent dyes or/and drugs. Isolated single cardiomyocytes have a rod-shaped form with a clear sarcomere striation pattern and preserved chamber-specific morphology as shown in Fig. 4. Obtained myocytes can be used within 8 hours of isolation prior measurements without membrane blebbing or loss of functional activity.

**Fig. 5:**
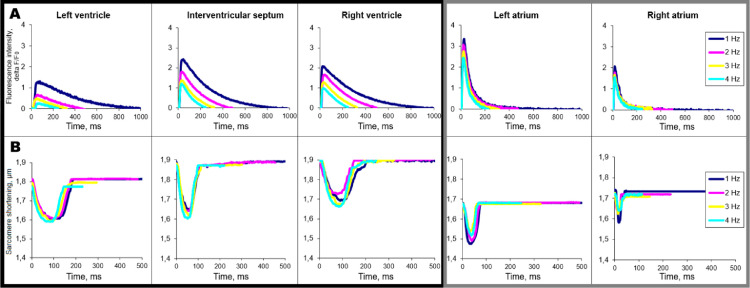
Representative recordings of cytosolic free calcium transient (A) and sarcomere shortening (B) in isolated ventricular (left panel, black frame) and atrial (right panel, gray frame) cardiomyocytes.

Observed data demonstrate pronounced regional differences in the amplitude and the time course of calcium transient and sarcomere shortening between the chambers consistently with [10], [11], [12] (Fig. 5). For ventricular cells, we observed rat-specific frequency-dependent modulation in the parameters of cytosolic calcium transient and sarcomere shortening. Both calcium transient decay as well as sarcomere relaxation are accelerated with increased pacing frequency as previously shown [13], [14], [15], [16]. Notably, the discrepancy between the frequency-dependent decrease in the amplitude of calcium transient and frequency-independency in the amplitude of sarcomere shortening is consistent with the literature data obtained from rats [17,18]. These results indicate normal contractile ability of obtained cardiomyocytes.

## Conclusions

The presented protocol provides simultaneous isolation of high yields of healthy viable cardiomyocytes from different heart chambers. It can be used for biophysical and biomechanical studies, notably in the area of myocardial heterogeneity research. The combination of Langendorff-based and Langendorff-free methods allows one to keep the advantages of each technique, such as the automation of the experimental protocol with a stable solution flow during the Langendorff retrograde perfusion and local perfusion control during the intraventricular and intra-atrial injections. Additionally, the use of solutions with different enzymatic activity ensures a high success rate for precise isolation of cells from different heart chambers.

Our protocol was developed taking into account the collagen content in rats. However, due to the simplicity and convenient control of the experimental manipulations, this protocol can be adapted for any species.

## Declaration of Competing Interest

The authors declare that they have no known competing financial interests or personal relationships that could have appeared to influence the work reported in this paper.
